# Geostatistical modelling of the distribution, risk and burden of podoconiosis in Kenya

**DOI:** 10.1093/trstmh/trac092

**Published:** 2022-09-21

**Authors:** Kebede Deribe, Hadley Matendechero Sultani, Collins Okoyo, Wyckliff P Omondi, Isaac Ngere, Melanie J Newport, Jorge Cano

**Affiliations:** Children's Investment Fund Foundation, Addis Ababa, Ethiopia; Brighton and Sussex Centre for Global Health Research, Department of Global Health and Infection, Brighton and Sussex Medical School, Brighton, UK; School of Public Health, College of Health Sciences, Addis Ababa University, Addis Ababa, Ethiopia; Kenya National Public Health Institute, Nairobi, Kenya; Eastern and Southern Africa Centre of International Parasite Control, Kenya Medical Research Institute, Nairobi, Kenya; Division of Vector Borne and Neglected Tropical Diseases, Ministry of Health, Nairobi, Kenya; Global Health Program, Washington State University, Nairobi, Kenya; Brighton and Sussex Centre for Global Health Research, Department of Global Health and Infection, Brighton and Sussex Medical School, Brighton, UK; Expanded Special Project for Elimination of Neglected Tropical Diseases, World Health Organization Regional Office for Africa, Brazzaville, Republic of the Congo

**Keywords:** geostatistical modelling, Kenya, podoconiosis, risk, spatial analysis

## Abstract

**Background:**

Understanding and accurately predicting the environmental limits, population at risk and burden of podoconiosis are critical for delivering targeted and equitable prevention and treatment services, planning control and elimination programs and implementing tailored case finding and surveillance activities.

**Methods:**

This is secondary analysis of a nationwide podoconiosis mapping survey in Kenya. We combined national representative prevalence survey data of podoconiosis with climate and environmental data, overlayed with population figures in a geostatistical modelling framework, to predict the environmental suitability, population living in at-risk areas and number of cases of podoconiosis in Kenya.

**Results:**

In 2020, the number of people living with podoconiosis in Kenya was estimated to be 9344 (95% uncertainty interval 4222 to 17 962). The distribution of podoconiosis varies by geography and three regions (Eastern, Nyanza and Western) represent >90% of the absolute number of cases. High environmental suitability for podoconiosis was predicted in four regions of Kenya (Coastal, Eastern, Nyanza and Western). In total, 2.2 million people live in at-risk areas and 4.2% of the total landmass of Kenya is environmentally predisposed for podoconiosis.

**Conclusions:**

The burden of podoconiosis is relatively low in Kenya and is mostly restricted to certain small geographical areas. Our results will help guide targeted prevention and treatment approaches through local planning, spatial targeting and tailored surveillance activities.

## Introduction

The World Health Organization (WHO) defines podoconiosis as a non-infectious tropical disease characterised by steadily progressive lymphoedema, often misdiagnosed as lymphatic filariasis (LF).^1^ Podoconiosis is believed to result from inflammatory processes triggered by barefoot exposure to irritant particles found in specific soil types derived from volcanic rock at high altitude that has been weathered in particular ways. This limits its occurrence to regions where these parameters exist. However, exposure to the necessary environmental conditions is not sufficient. There is strong evidence for a genetic association with class II human leucocyte antigen (HLA) genes. Even then, podoconiosis only develops in vulnerable individuals due to socio-economic deprivation and poor access to foot hygiene and shoes.^1–3^ It is one of the leading causes of lymphoedema in Africa.^4^ The disease significantly reduces quality of life^5^ and productivity through decreased mobility and associated morbidity. People with podoconiosis suffer from mental distress and depression, driven by stigma and discrimination.^6,7^

Podoconiosis is amenable to prevention through simple public health interventions^3,8^ such as consistent use of footwear from an early age, regular foot hygiene and covering housing floors.^9^ For those with the disease, the WHO recommends hygiene-based lymphoedema management, which includes foot hygiene, foot care, wound care, compression, exercises and foot elevation, treatment of ‘acute attacks’ (painful episodes of an inflammatory condition known as acute dermatolymphangioadenitis) and use of shoes and socks to reduce further exposure to the irritant soil.^8^

Global figures indicate that 4 million people live with podoconiosis in 27 countries, mainly in the highland areas of tropical Africa, Latin America and South East Asia.^10^ Only three countries (Cameroon, Ethiopia and Rwanda) have mapped the distribution of podoconiosis through nationwide surveys, which have revealed a widespread distribution of podoconiosis and a subsequent higher burden of disease than initially expected.^11–13^ These studies have also increased our knowledge of the environmental, climatic and soil composition–related factors associated with the occurrence of podoconiosis.

Accurate characterisation of the environmental conditions associated with the occurrence of podoconiosis (which we refer to as environmental limits) combined with an understanding of the population at risk and the burden of disease are critical for delivering targeted and equitable prevention and treatment services. This information also allows planning of control and elimination programs, tailored case finding and disease surveillance. Epidemiological data are typically obtained through conducting house-to-house case searches or large-scale surveys. Kenya has recently conducted a countrywide survey to determine the geographical distribution of podoconiosis and identify individual and village-level risk factors associated with its occurrence.^14^ Although such data are important, they do not include environmental data that could provide high spatial resolution or reliable measures of uncertainty to support effective decision making and planning. Therefore, using national data and building on previous modelling work, we undertook a secondary analysis to predict the environmental limits and estimate the prevalence and number of cases of podoconiosis in Kenya. The current analysis results will contribute to national efforts to eliminate this public health issue by informing targeted and equitable access to prevention and care.

## Methods

### Podoconiosis prevalence data

In 2019, a nationwide population-based cross-sectional survey in 48 villages in 24 subcounties across 15 counties covering the Western, Nyanza, Eastern, North Eastern, Rift Valley and Coast regions of Kenya was conducted. Two villages in each subcounty were included in the study and the target population was residents of the selected villages. In each selected village, 50 households were selected using a systematic random sampling technique, the details of which are published elsewhere.^14^

### Explanatory environmental variables

A collection of >50 remotely sensed environmental datasets including climate, soil-related, topographic, vegetation density and urbanicity level data previously identified as potential risk factors for the occurrence of podoconiosis were assembled and used to ascertain the environmental limits of the disease in Kenya.^15,16^ Using principal component analysis to reduce dimensionality and eliminate correlated variables, we selected environmental datasets that best characterise the environment at locations where podoconiosis cases had been diagnosed during the nationwide cross-sectional survey: mean temperature of the wettest quarter, precipitation of the coldest quarter, concentration of extractable iron content (mg/kg soil), percentage of orthent soils, distance to water bodies, flow accumulation and night-light (NL) emissivity are available in the appendix. Geographic coordinates of each surveyed villages were used to extract pixel values from gridded maps of the selected datasets.

Information on precipitation and temperature were extracted from a synoptic gridded map of annual precipitation calculated from monthly total precipitation gridded datasets obtained from the WorldClim database (version 2.1).^17^ This database provides a set of global climate layers obtained by interpolation of precipitation data for the period 1970–2000 collected in weather stations distributed across the world.^18,19^ We obtained a raster elevation dataset at 1 km from the Consortium for Spatial Information.^2,20^ This elevation layer resulted from processing and resampling the gridded digital elevation models (DEMs) derived from the original 30-arcsecond DEM produced by the Shuttle Radar Topography Mission. Flow accumulation was derived from the elevation raster dataset. The flow accumulation surface represents each cell's potential to accumulate water. This was generated via a flow direction raster that identifies the direction of flow as the steepest descent from each cell in the elevation dataset, calculated as change in elevation/distance*100. The flow accumulation raster was derived by summing the flow direction value weights of all cells predicted to flow into each cell. We also generated continuous surfaces of straight-line distance (Euclidean distance) in kilometres to the nearest water body based on updated maps of waterbodies and waterways from Kenya downloaded from the OpenStreetMap project^21^ through the platform Geofabrik.^22^

Soil data including the concentration of extractable iron (mg/kg of topsoil) and predicted distribution for the orthent soil class were obtained from the International Soil Reference and Information Centre World Soil Information project^23,24^ and the OpenGeoHub project,^25^ respectively. These projects provide gridded maps of soil composition at 250 m resolution worldwide. Orthent soils are a suborder of the entisol soil type characterized by their extreme shallowness, steepness and consequent high erosion hazard. They are poor soils and therefore not suitable for farming. In Africa, orthents occur in flat terrain because the parent rock contains no weatherable mineral except short-lived additions from rainfall.

Finally, we obtained a raster of stable NL emissivity in 2010 (the median year of detection of the included cases) from the National Oceanic and Atmospheric Administration.^26^ The Operational Linescan System instrument, on board a satellite of the Defence Meteorological Satellite Programme, measures visible and infrared radiation emitted at night, resulting in remote imagery of lights on the ground. This information has been correlated with gross domestic product in developed countries^27,28^ and, although far from being precise, provides an indirect measure of poverty in developing countries.^29^ NL emissivity is provided as gridded maps of 1 km^2^ resolution, with values ranging from 0 (undetectable NL emissivity) to 60 (maximum NL emissivity).

Input grids were resampled to a common spatial resolution of 1 km^2^ using the nearest neighbour approach, clipped to match the geographic extent of the map of Kenya and eventually aligned to it. Raster manipulation and processing was undertaken using the raster package in R version 3.6.3 (R Foundation for Statistical Computing, Vienna, Austria) and final map layouts created with ArcGIS 10.7 software (ESRI, Redlands, CA, USA).

### Environmental modelling using regression-based and machine learning algorithms

Villages surveyed in the nationwide mapping were reclassified as endemic (1) or non-endemic (0) for podoconiosis based on records of confirmed podoconiosis cases. This reported occurrence of podoconiosis in the surveyed villages and the selected environmental factors described above were used to model the distribution of podoconiosis in relation to the environmental variables using various algorithms that were developed previously to predict the distribution of species across geographical spaces (e.g. for animal or plant conservation studies). We used seven types of algorithms, of which three were linear regression based algorithms and four were machine learning algorithms, available within the BIOMOD (BIOdiversity MODelling) framework, an established analytical framework developed to model species distribution.^30,31^ These algorithms were generalized linear models (GLMs), generalized additive models, generalized boosted regression trees models (BRTs), artificial neural networks, multiple adaptive regression splines, maximum entropy (MaxEnt) and random forest (RF). All the algorithms except the BRTs were run using the parameters set by default in the biomod2 R package.^30^ For the BRT algorithm, the learning rate (lr) and tree complexity (tc) were set, enabling the model to account for up to four potential interactions and slowing it down enough (lr=0.005) to get the model converged without overfitting the data. This tuning was undertaken using the gbm package in R version 3.6.3. Different combinations (technically known as ensembles) of the algorithms were used to explore which model gave the best fit to the data.

All these models were intended to discriminate the suitability of the environment for the presence of podoconiosis (i.e. environmental suitability). For this, they needed to be trained with presence and absence records. In addition to the recorded presences and absences, we generated a set of pseudoabsence points representing areas presumably unsuitable for podoconiosis. The use of pseudoabsence points to represent areas presumed to be unsuitable for a species is a well-established approach in species distribution modelling.^32,33^ We implemented pseudoabsence selection using the ‘surface range envelope’ approach to define the area of assumed unsuitability. The envelope is estimated through a presence-only suitability model^34^ that identifies the range of locations at which the values of the chosen environmental covariates are within a specified range (here between the 5th and 95th percentiles) of the covariate values at the occurrence locations.^3^^0^ Five sets of 500 pseudoabsence samples were randomly extracted from outside this envelope. Every set of pseudoabsence was pulled together with the presence and absence records and used with each algorithm to construct a single model. Models (50 models per algorithm totalling 350 ensembles) were calibrated using an 80% random sample of the initial data and evaluated against the remaining 20% of data using the area under the curve (AUC) of the receiver operating characteristics (ROC) curve, the true skill statistic (TSS)^35^ and the proportion correctly classified (PCC). The evaluation statistics (AUC and TSS) were used to select the models to be assembled based on the matching between predictions and observations. Here, models with an AUC <0.8 were disregarded when assembling the final model.

The final ensemble model was obtained by estimating the weighted mean of probabilities across the selected models per grid cell. This algorithm returned the predicted mean weighted by the selected evaluation method scores, in our case the AUC statistic score. The range of uncertainties was also calculated by estimating the uncertainty intervals around the mean of probabilities across the ensemble per grid cell. The resulting predictive map quantified the environmental suitability for podoconiosis. In order to convert this continuous metric into a binary map outlining the distribution limits, a threshold value of suitability was determined, above which occurrence of podoconiosis was assumed to be possible. The ROC curve determined the threshold value that represents a better trade-off between sensitivity, specificity and PCC.

In addition, partial dependence functions were performed separately for BRT-based models to visualise dependencies between the probability of podoconiosis occurrence and covariates. The partial dependence function shows the marginal effect of each covariate on the response after averaging the effects of all other covariates.

### Geostatistical modelling to estimate disease burden

Village-level prevalence data and resulting environmental suitability values were then used within a geostatistical framework. We developed a geostatistical model to predict podoconiosis prevalence in areas where the occurrence of podoconiosis was predicted, using the environmental modelling results, at the village level across Kenya. We let podoconiosis risk depend on the predicted environmental suitability value obtained in the previous step. We included spatial random effects to account for spatial variation in podoconiosis prevalence between villages that is not explained by the explanatory variable. We validated the model using a variogram-based procedure that tests the compatibility of the adopted spatial structure with the data. More details are provided in the appendix. The analysis was carried out using the PrevMap R package,^36^ which implements parameter estimation and spatial prediction of geostatistical models. This model was applied to produce continuous predictions of prevalence of podoconiosis among adults (≥15 y of age) at 1-km^2^ spatial resolution and probability maps of exceeding a 1% prevalence threshold, which was used to define podoconiosis endemicity. We checked the validity of the assumed covariance model for the spatial correlation using the Monte Carlo algorithm and empirical semi-variogram as described in the supplemental file. Additionally, maps of the number of standard errors from the posterior mean prevalence of podoconiosis and number of cases were generated for each 1 km × 1 km grid location.

Gridded maps of both population density and age structure were obtained from the WorldPop project.^37,38^ We used these gridded surfaces of population estimates to compute the potential affected adult population (≥15 y of age). An output raster dataset computing the estimated number of podoconiosis cases per grid cell was obtained by multiplying the 1-km^2^ raster dataset of predictive prevalence with the corresponding adult population density surface. The same procedure was used to estimate the uncertainty range of the affected population using the gridded surfaces of the 95% uncertainty interval (UI) for predicted prevalence. These surfaces were then used to extract the aggregate number of people with podoconiosis and the uncertainty range by administrative area (subcounties and counties). In brief, the 95% UI was calculated based on the uncertainty in environmental suitability, by summarising the 50 predictions by mean and 95% credible intervals.

## Results

### Main outcomes of the survey

**Figure 1.**
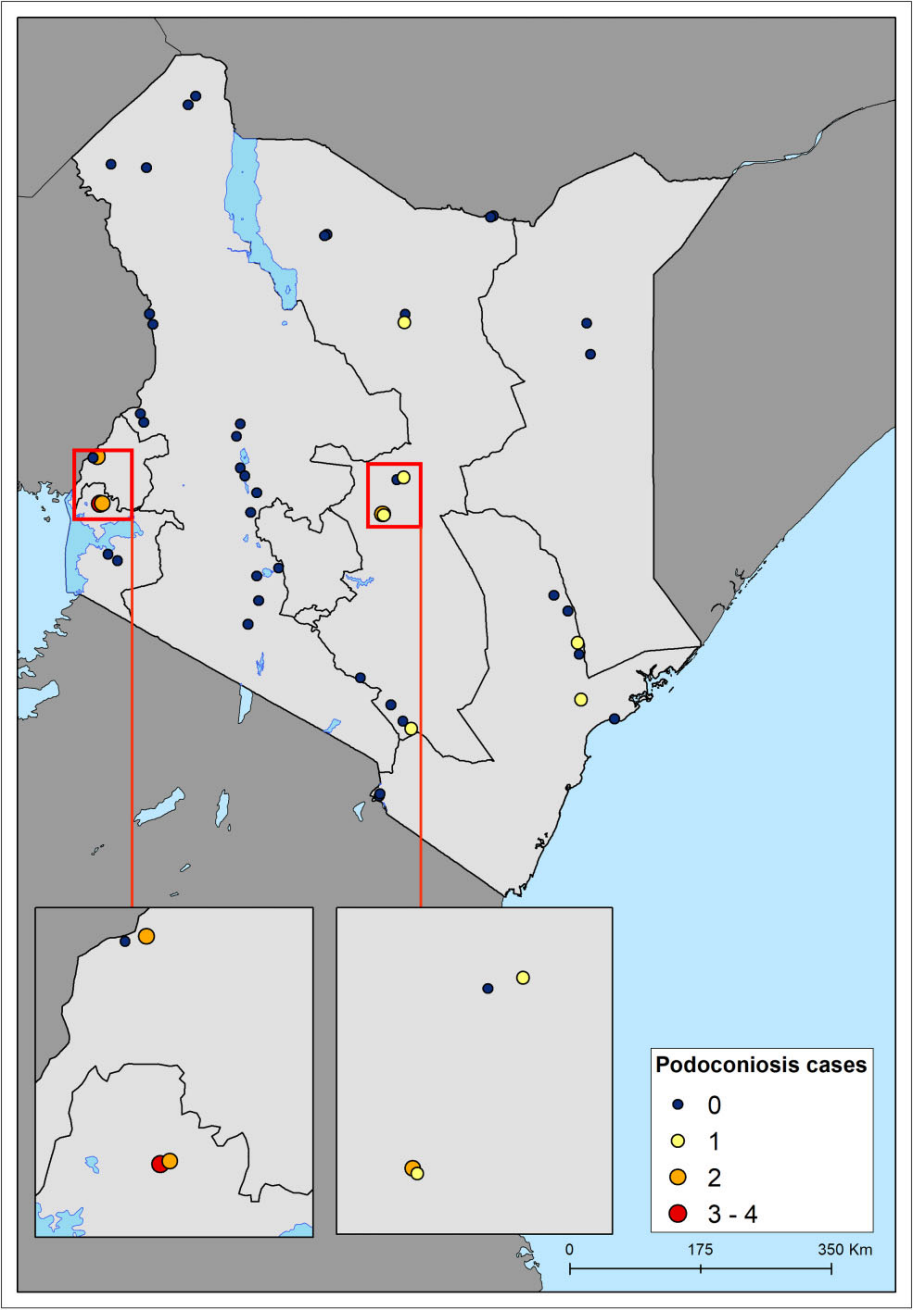
Distribution of surveyed villages and number of cases of podoconiosis recorded.

In 2019, a national survey was conducted in Kenya in 48 villages in 24 subcounties across 15 counties covering the Western, Nyanza, Eastern, North Eastern, Rift Valley and Coast regions of Kenya. In each village, an average of 43 households (range 12–120) and 129 participants (range 44–311) were surveyed. Overall, data were collected from 2024 households and 6228 participants. Overall, 16/6228 (0.3% [95% confidence interval 0.1 to 0.5]) of the participants were diagnosed with podoconiosis. Analysis by county indicated that podoconiosis cases were prevalent in six counties: Siaya, Meru, Busia, Makueni, Marsabit and Tana River. Accordingly, analysis of prevalence by subcounty revealed that podoconiosis cases were prevalent in eight subcounties. Of the 48 surveyed villages, 10 reported at least one case of podoconiosis (range one to four) (Figure 1).

### Factors associated with podoconiosis occurrence

Figures in the appendix show the marginal effect of each covariate on the probability of podoconiosis occurrence, while the relative contribution of each predictor variable on the outcome (podoconiosis prevalence) is summarised in the supplementary file (Figure 4S). The covariate contribution was estimated separately for the BRT and RF models. Of the selected seven covariates, three variables (iron content, probability of having an orthent-type soil and mean temperature of the wettest quarter) appeared to be the major contributors to both the BRT and RF models. When the extractable iron content exceeded 100 mg/kg, the probability of podoconiosis occurrence increased. The probability of having an orthent-type soil was negatively associated with the probability of podoconiosis occurrence. There was a higher risk of podoconiosis occurrence when the mean temperature during the wettest quarter was 20°C–25°C. The presence of podoconiosis became increasing more likely the closer the land was to water, and it was particularly high in areas with steep slopes.

### Environmental limits of podoconiosis in Kenya

**Figure 2.**
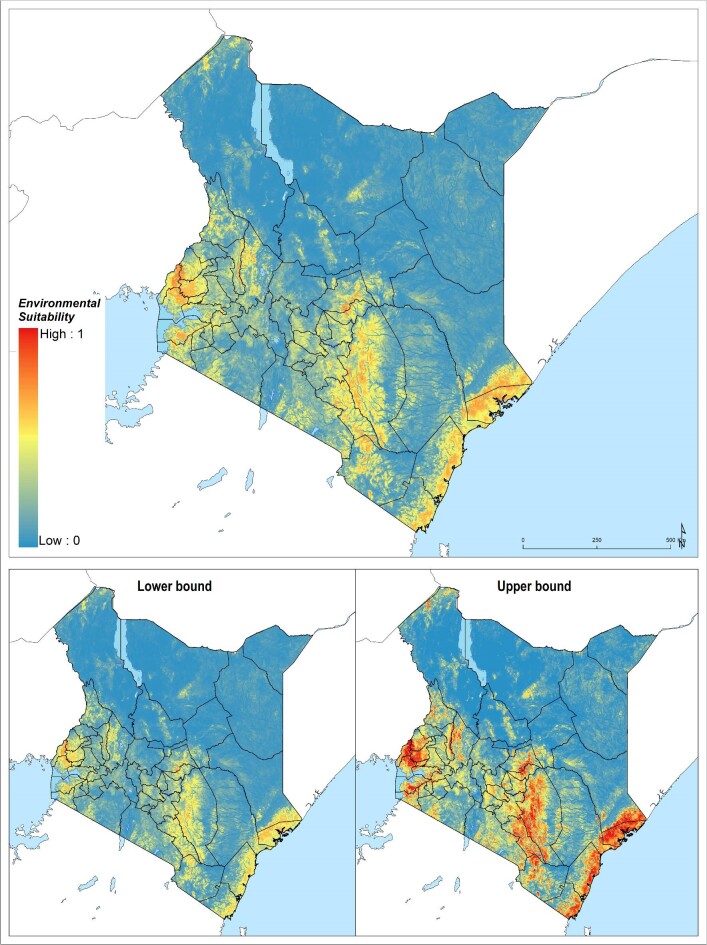
Ensemble of predicted environmental suitability models for podoconiosis and corresponding uncertainty of prediction. Uncertainty was calculated as the range of the 95% UI in predicted probability of occurrence for each pixel and rescaling to a 0–1 scale.

In total, 4.2% of the landmass of Kenya was found to be environmentally suitable for the occurrence of podoconiosis. Most of the land mass suitable for the occurrence of podoconiosis was situated in the Coastal, Eastern and Nyanza regions (Table 1, Figure 2). A total of 2.2 million people live in an environment suitable for the occurrence of podoconiosis, the majority of which were from the Coastal, Eastern, Nyanza and Western regions.

**Table 1.** Estimation of podoconiosis cases by regions in Kenya

			Estimated podoconiosis cases		
				95% CI	
Province	Predicted suitable area (sq. km)	Population living in suitable areas	n	Lower bound	Upper bound
Central	387	38 805	25	11	49
Coast	8817	270 203	816	353	1612
Eastern	7003	468 451	1112	495	2160
Nairobi	–	–	–	–	–
Northeastern	2185	2539	8	3	16
Nyanza	2153	510 888	2302	1061	4373
Rift Valley	1211	36 090	29	14	56
Western	2649	912 192	5052	2285	9696
Total	24 405	2 239 168	9344	4222	17 962

### Validation statistics

Validation statistics indicated an excellent predictive performance of all the algorithms (see the Appendix). However, the BRT, MaxEnt and GLM performed better than the other models, with AUC scores of 0.83 (95% UI 0.75 to 0.94), 0.84 (95% UI 0.82 to 0.92) and 0.82 (95% UI 0.71 to 0.96), respectively. An environmental suitability threshold of 0.602 provided the best discrimination between presence and absence records, with a sensitivity of 100%, specificity of 98.53% and AUC score of 0.996. This threshold value was used to classify the environmental suitability map into a binary map of the environmental limits of occurrence, which is included in the Appendix. The variogram fitted on the residuals of the modelled prevalence leads us to conclude that the data were compatible with the assumptions of an exponential correlation function and that the underlying spatial structure was accounted for by the spatial fixed and random effects (see Appendix).

### Predicted prevalence and estimation of podoconiosis burden

**Figure 3.**
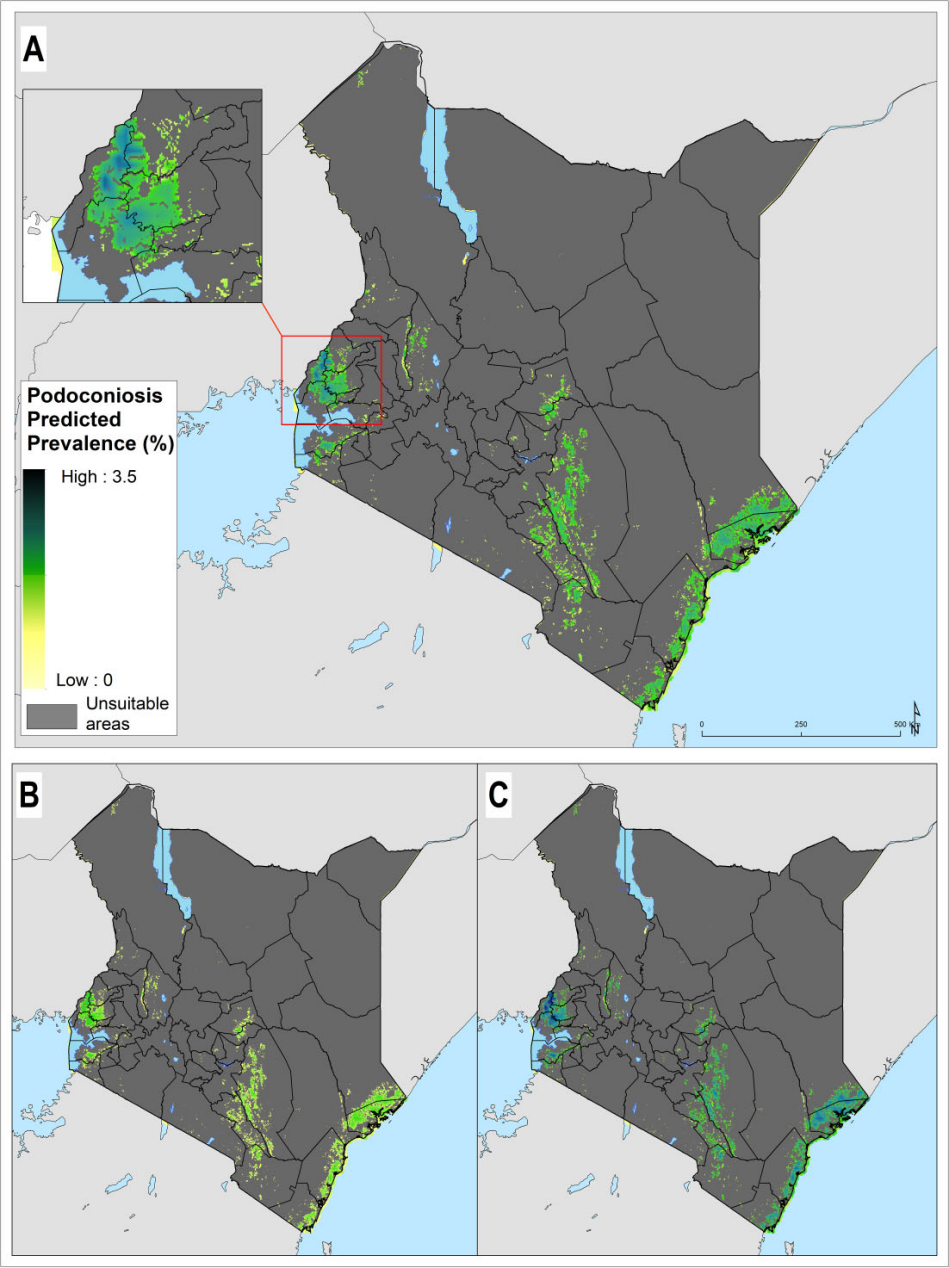
Predicted podoconiosis prevalence maps of Cameroon. (A) Mean predicted prevalence and (B) lower and (C) upper 95% UI bounds. Areas considered environmentally unsuitable for the occurrence of podoconiosis as predicted by the environmental model have been excluded.

**Figure 4.**
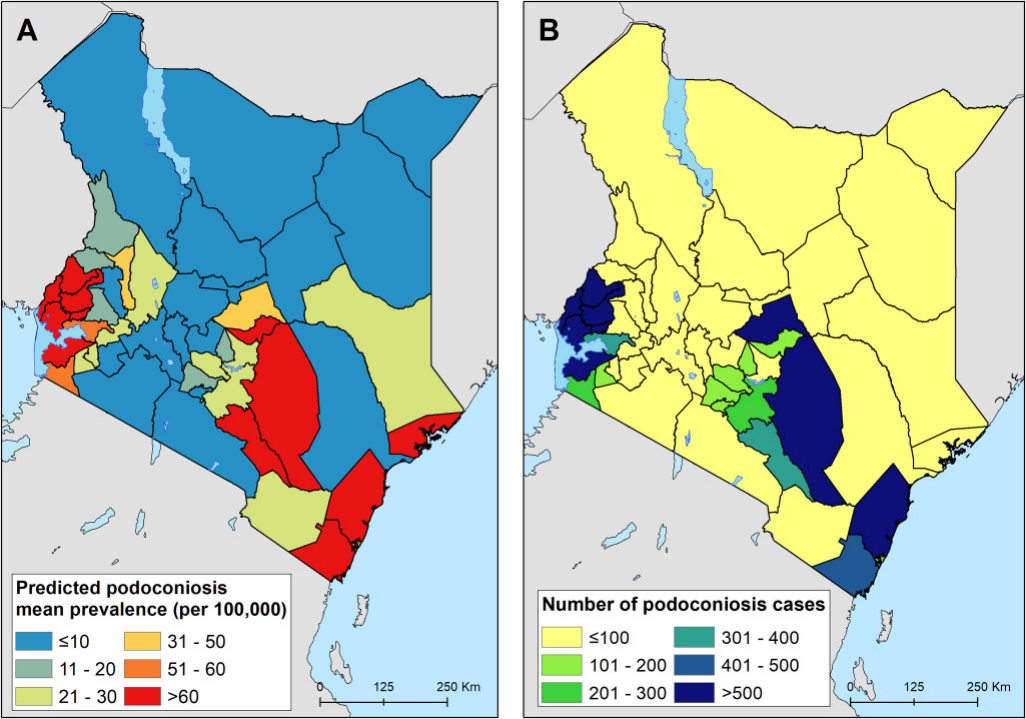
County-level predicted prevalence of podoconiosis. (A) Mean predicated prevalence of podoconiosis and (B) estimated number of people with podoconiosis.

**Figure 5.**
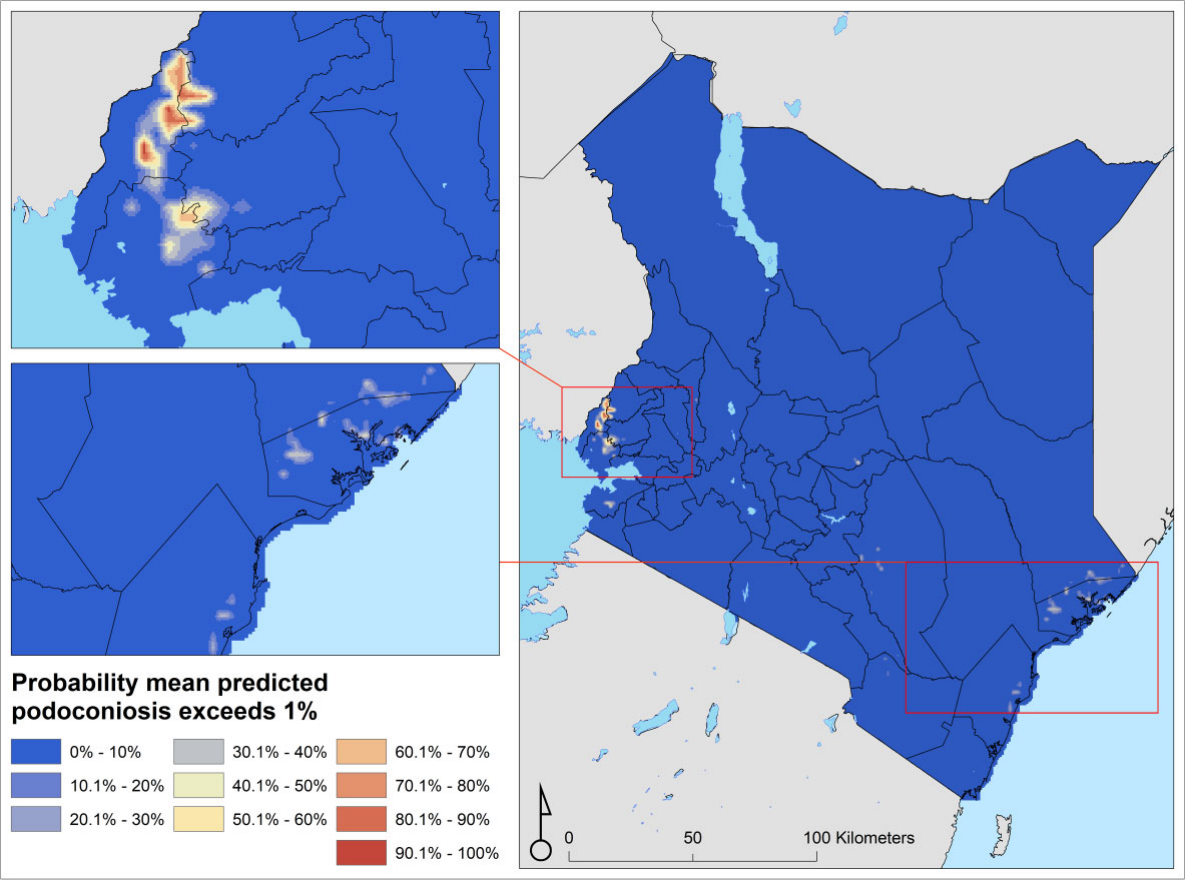
Map of probability of exceeding 1% podoconiosis prevalence in Kenya.

The prevalence of podoconiosis was predicted to be variable in four regions (Coastal, Eastern, Nyanza and Western) (Figure 3). In the remaining regions, the distribution of podoconiosis was predicted to be focal and of low prevalence. Podoconiosis cases were prevalent in pockets of villages in the western and central parts of Kenya. Nationally, we estimated 9344 people (95% UI 4222 to 17 962) to be living with podoconiosis in 2020 in Kenya (Table 1). Three regions (Eastern, Nyanza and Western) contributed to >90% of the absolute number of cases. The greatest proportion (54%) of people with podoconiosis resided in the Western region, surrounding Lave Victoria in the Kisumu area. At least one case of podoconiosis was predicted in 33 of the 47 counties in Kenya. Eleven counties were predicted to have >100 cases of podoconiosis, while only six had >500 predicted cases (Figure 4 and Appendix). We also estimated the continuous probability of exceeding 1% podoconiosis prevalence (the threshold considered for intervention) across the endemic areas (Figure 5). Most areas showed a low probability of exceeding 1%, and only a few restricted areas of the Coastal and Western regions potentially exceeded that threshold.

## Discussion

This secondary analysis aimed to determine the environmental limits to the distribution of podoconiosis and estimate its burden in Kenya to provide evidence to inform the Ministry of Health and WHO action plans for the prevention, management and elimination of podoconiosis.^1^ Several regions in Kenya were found to have environments suitable for the development of podoconiosis. The number of cases in these regions ranged from 25 to 5052. The environmental extent, population at risk and number of cases of podoconiosis in the country is small compared with other endemic countries, making the elimination of podoconiosis feasible with concerted effort to expand prevention and case management interventions.

The predicted number of cases of podoconiosis in Kenya (9344) was lower than what was estimated in Cameroon (41556)^12^ and Ethiopia (1.56 million)^11^ but higher than the estimate in Rwanda (6429).^13^ In addition, the areas suitable for podoconiosis were geographically restricted in Kenya (i.e. 4.2% of the total landmass of Kenya) compared with Ethiopia (24%).^16^ The burden of podoconiosis coupled with almost universal shoe wearing at an early age implies that with little effort to scale up the prevention and management of podoconiosis, the country is poised to eliminate podoconiosis.^14^ Increased urbanization, improved access to water and infrastructure development, including improved housing and road construction, will pay dividends in reducing the burden of podoconiosis and ultimately its elimination.

The prevalence and burden of podoconiosis in Kenya is geographically variable. There was no risk or cases predicted in the Nairobi region. This agrees with this region's better socio-economic and infrastructure development compared with other regions. A high burden is estimated in the Eastern, Nyanza and Western regions. The Eastern region is a lowland area with a lot of mining exploration and sand harvesting, most often done when people work barefooted. The Nyanza and Western regions are largely highlands and mountainous, they receive relatively high amounts of rainfall^39,40^ and the soil types in these areas are largely volcanic, which are thought to contain irritant minerals that can trigger inflammatory processes.^41,42^ Interventions targeted towards podoconiosis should prioritize these regions. The number of podoconiosis cases per county is small in most counties. Only 6 counties had >500 cases and only 11 counties had >100 cases. This implies that the morbidity management of podoconiosis can easily be integrated at dispensaries, health centres and subcounty hospitals as part of routine health services without the need to set up a stand-alone podoconiosis control program.^43^ More than 60% of the cases were predicted in three counties (Kakamega, Siaya and Busia). Prioritising these high-burden counties would help significantly reduce the burden of podoconiosis and advance the national goal of elimination.

Although at a low prevalence, podoconiosis is documented in Kenya's Coastal region. Previous studies documented that the region is endemic for LF.^44,45^ This implies that the region is where podoconiosis and LF overlap geographically, as described elsewhere.^4^ Therefore, health workers in the region should be trained to have a high index of suspicion for lymphoedema due to these two diseases. Even if the morbidity management is similar for the two diseases, there is a need to differentiate the cause of lymphoedema to tailor preventive public health education and social mobilization strategies based on the aetiology of lymphoedema in the region.

Our modelling approach is not without limitations. First, the number of data points used in the analysis is minimal (i.e. 48 villages). Nonetheless, the data points were geographically distributed across Kenya. We believe the data points captured the different geographic, climatic, spatial variability and environmental characteristics to accurately define the geographical limits of podoconiosis in Kenya. Second, an ongoing challenge for podoconiosis modelling is the absence of covariates at the required spatial scale.^4,12,13^ This includes access to shoes and shoe-wearing practices. Host genetic factors are also determinants of susceptibility to podoconiosis, but the mechanisms are currently poorly understood beyond the clear association with class II HLA gene variation. HLA genes are highly polymorphic and the frequencies of the different gene variants vary considerably between populations. Therefore it is impossible to extrapolate findings from other endemic populations to Kenyan populations, so this information could not be included in our modelling work. Third, limitations exist in the survey data that were used to construct the models. Data quality issues such as sampling bias may have arisen when remote areas were left out due to inaccessibility. In addition, underestimation of podoconiosis cases might have resulted from lack of mobility and associated stigma.^14^ Looking to the future, as additional covariates and prevalence data continue to be collected, it will be important to extend this modelling framework to include shoe-wearing practices, the poverty index and genetic susceptibility to podoconiosis. This will help to include individual behaviours data in addition to climatic and environmental data in the models.

Our analysis provided important insights into the geographical distribution, environmental limits and burden of podoconiosis in Kenya. Such information is critical for tackling the disease, designing preventive interventions and monitoring progress. We identified the high-risk areas and high-burden counties where the focus of prevention and morbidity management interventions should be. Such information helps the Ministry of Health determine where to prioritize resources and efforts to bring about high impact and value for the money at an early intervention stage.

In conclusion, our analysis has identified restricted geographical and environmental suitability for podoconiosis in Kenya. In addition, the number of estimated cases in the country compared with other countries is low. Most of the cases were found in three counties: Kakamega, Siaya and Busia. Therefore, an approach targeting these three high-burden counties would be an efficient way of planning podoconiosis prevention and treatment interventions. The findings also suggest that the Kenyan Ministry of Health should plan and roll out a podoconiosis response, including morbidity management, footwear use at an early age and foot hygiene practices. The rollout of interventions can be achieved by integrating these interventions and services within the national health structure, focusing on dispensaries, health centres and subcounty hospitals. There is a need for inclusion of podoconiosis in the community-based surveillance system. Intensified and tailored behavioural change communication and social transformation is required to address the preventable root causes of podoconiosis (barefoot and poor foot hygiene practices), which will advance the national goal and accelerate the progress towards a world without podoconiosis.

## Supplementary Material

trac092_Supplemental_File

## Data Availability

The data used in this study are published in this article and supplementary material.
