# Supplementary material for: Geostatistical modelling of the distribution, risk and burden of podoconiosis in Kenya
Source: Trans R Soc Trop Med Hyg. 2022 Sep 21;117(2):72–82. doi: 10.1093/trstmh/trac092 (PMC9890307; doi:10.1093/trstmh/trac092)
Supplement: Supplementary file 1 [file trac092_supplemental_file.docx]

**Supplementary Appendix** 

**Modelling the distribution, risk and burden of podoconiosis in Kenya** 

Kebede Deribe Ph.D.^1,2,3^, Hadley Matendechero Sultani M.D.^4^, Collins Okoyo M.Sc.^5^, Wyckliff P. Omondi M.Sc.^6^, Isaac Ngere M.D.^7^, Melanie J. Newport Ph.D.^2^ and Jorge Cano Ph.D.^8^ 

1. Children’s Investment Fund Foundation, Addis Ababa, Ethiopia 

2. Brighton and Sussex Centre for Global Health Research, Department of Global Health and Infection, Brighton and Sussex Medical School, Brighton, United Kingdom

3. School of Public Health, College of Health Sciences, Addis Ababa University, Addis Ababa, Ethiopia

4. Kenya National Public Health Institute, Nairobi, Kenya 

5. Eastern and Southern Africa Centre of International Parasite Control (ESACIPAC), Kenya Medical Research Institute (KEMRI), Nairobi, Kenya

6. Division of Vector Borne and Neglected Tropical Diseases, Ministry of Health, Nairobi, Kenya

7. Global Health Program, Washington State University, Nairobi, Kenya

8. Expanded Special Project for Elimination of Neglected Tropical Diseases (ESPEN), World Health Organization’s Regional Office for Africa, Brazzaville, Republic of the Congo

**Text 1S. Formulation and validation of geostatistical model of podoconiosis prevalence** 

Let *Y*_*i*_ denote the number of positively tested podoconiosis cases at location *x*_*i*_ out of *n*_*i*_ sample individuals. We then assume that, conditionally on a zero-mean spatial Gaussian process *S(x)*, the *Y*_*i*_ are mutually independent Binomial variables with probability of testing positive *p(x*_*i*_*)* such that

$log\{\frac{p(x_{i})}{1-p(x_{i})}\}= β_{0}+ β_{1}EnvSuit(x_{i})+ S(x_{i})$

where the explanatory in the above equation is the modelled environmental suitability at location *x*_*i*_. 

We model the Gaussian process *S(x)* using an isotropic and stationary exponential covariance function given by 

$Cov\{S(x), S(x')\} =     σ^{2}exp\{-\vert\vert x-x'\vert\vert/ϕ\}$

Where $\vert\vert x-x'\vert\vert$is the Euclidean distance between *x* and *x’,*  $σ^{2}$ is the variance of *S(x)* and $ϕ$ is a scale parameter that regulates how fast the spatial correlation decays to zero for increasing distance. 

To check the validity of the adopted exponential correlation function for the spatial random effects *S(x)*, we carried out the following Monte Carlo algorithm.

1. Simulate a binomial geostatistical dataset at observed locations *x*_*i*_ by plugging-in the maximum likelihood estimates from the fitted model.

2. Estimate the unstructured random effects *Z*_*i*_ from a non-spatial binomial mixed model obtained by setting *S(x) =0* for all locations *x*.

3. Use the estimates for *Z*_*i*_ from the previous step to compute the empirical variogram.

4. Repeat steps 1 to 3 for 10,000 times. 

5. Use the resulting 1,000 variograms to compute the 95% tolerance bandwidth under the hypothesis that the analysed data were generated by the fitted model. If the empirical variogram from the original data, obtained as in step 2, lies within 95% bandwidth, we then conclude that we do not find evidence against the assumption of an exponential correlation function for *S(x)*.

**Figure 1S.** Maps of covariates used to model environmental suitability.

**Figure 2S Partial dependence plots of the relative contribution of mean temperature of wettest quarter, precipitation of coldest quarter and night-light emissivity to the boosted regression tree (BRT) model for podoconiosis, averaged over 50 ensembles.** Blue lines represent the mean partial dependence over all 50 BRT ensembles and grey envelopes the standard deviation from the mean. The *y-*axis is the transformed logit response and *x-*axis is the full range of covariates values. 

**Figure 3S Partial dependence plots of the relative contribution of topography (flow accumulation), distance to water bodies and soil composition (iron content and fraction of orthents type soil) related covariates to the boosted regression tree (BRT) model for podoconiosis, averaged over 50 ensembles.** Blue lines represent the mean partial dependence over all 50 BRT ensembles and grey envelopes the standard deviation from the mean. The *y-*axis is the transformed logit response and *x-*axis is the full range of covariates values. 

**Figure 4S. Variable contribution of final ensemble models based on** ***boosted regression trees***  **and** ***random forest*****.** Variable contribution is provided as percentage, and it shows the relative contribution of selected environmental predictors to the final ensemble model of predicted podoconiosis occurrence.

**Figure 5S.** **Predicted occurrence of podoconiosis** **and uncertainty range across Kenya.** This binary maps were generated from the predicted environmental suitability based on a cut-off above which the podoconiosis occurrence is highly likely. Optimal threshold was fitted to get better trade-off between sensitivity, specificity and proportion correctly classified (PCC).

**Figure 6S.** **The results of the Monte Carlo validation procedure.** The solid line is the observed variogram and the shaded area corresponds to the 95% bandwidth. The results lead us to conclude that the data are compatible with the assumption of an exponential spatial correlation function.

## **Table 1S. Validation statistics for the algorithms used to construct the environmental suitability model.** Generalized linear models (GLM), generalized additive models (GAM), generalized boosted regression trees models (BRT), artificial neural networks (ANN), multiple adaptive regression splines (MARS), maximum entropy (MaxEnt) and random forest (RF).  

|  | **GLM** | **GAM** | **GBM** | **ANN** | **MARS** | **RF** | **MAXENT** |
| --- | --- | --- | --- | --- | --- | --- | --- |
| **ROC Mean** | 0.818 | 0.644 | 0.834 | 0.738 | 0.597 | 0.749 | 0.844 |
| **ROC Median** | 0.898 | 0.728 | 0.865 | 0.765 | 0.566 | 0.787 | 0.875 |
| **ROC Lower Bound** | 0.710 | 0.486 | 0.748 | 0.613 | 0.471 | 0.659 | 0.819 |
| **ROC Upper Bound** | 0.960 | 0.740 | 0.938 | 0.881 | 0.709 | 0.848 | 0.920 |

## **Table 2S. Estimation of podoconiosis cases by county in Kenya** 

| **Province** | **County** | **Area predicted suitable (sq-km)** | **Adult Population living in suitable areas** | **Estimated Podoconiosis Cases** |  |  |
| --- | --- | --- | --- | --- | --- | --- |
|  |  |  |  | **No.** | **95% CI** |  |
|  |  |  |  |  | **Lower Bound** | **Upper Bound** |
| Central | Kiambu | 2 | 763 | 0 | 0 | 0 |
| Central | Kirinyaga | 43 | 20,092 | 14 | 6 | 27 |
| Central | Murang'a | 27 | 12,057 | 6 | 3 | 13 |
| Central | Nyandarua | 0 | 0 | 0 | 0 | 0 |
| Central | Nyeri | 1 | 259 | 0 | 0 | 0 |
| Coast | Kilifi | 2,357 | 168,888 | 553 | 240 | 1,095 |
| Coast | Kwale | 1,165 | 70,793 | 203 | 88 | 401 |
| Coast | Lamu | 3,563 | 12,646 | 35 | 15 | 70 |
| Coast | Mombasa | 5 | 427 | 1 | 0 | 1 |
| Coast | Taita Taveta | 1,128 | 6,313 | 6 | 2 | 11 |
| Coast | Tana River | 599 | 11,136 | 18 | 8 | 34 |
| Eastern | Embu | 44 | 8,920 | 9 | 4 | 18 |
| Eastern | Isiolo | 27 | 359 | 0 | 0 | 0 |
| Eastern | Kitui | 4,237 | 184,831 | 578 | 252 | 1,141 |
| Eastern | Machakos | 174 | 17,164 | 25 | 11 | 49 |
| Eastern | Makueni | 1,561 | 91,387 | 146 | 64 | 286 |
| Eastern | Marsabit | 3 | 33 | 0 | 0 | 0 |
| Eastern | Meru | 657 | 133,932 | 282 | 131 | 528 |
| Eastern | Tharaka-Nithi | 300 | 31,825 | 72 | 33 | 138 |
| Nairobi | Nairobi | 0 | 0 | 0 | 0 | 0 |
| North Eastern | Garissa | 2,185 | 2,539 | 8 | 3 | 16 |
| North Eastern | Mandera | 0 | 0 | 0 | 0 | 0 |
| North Eastern | Wajir | 0 | 0 | 0 | 0 | 0 |
| Nyanza | Homa Bay | 638 | 113,495 | 365 | 159 | 723 |
| Nyanza | Kisii | 53 | 21,398 | 31 | 14 | 62 |
| Nyanza | Kisumu | 202 | 48,33 | 86 | 39 | 167 |
| Nyanza | Migori | 303 | 49,959 | 67 | 29 | 134 |
| Nyanza | Nyamira | 29 | 13,515 | 17 | 8 | 34 |
| Nyanza | Siaya | 928 | 264,188 | 1,736 | 812 | 3,253 |
| Rift Valley | Baringo | 504 | 7,997 | 8 | 3 | 15 |
| Rift Valley | Bomet | 6 | 1,837 | 1 | 1 | 2 |
| Rift Valley | Elgeyo-Marakwet | 324 | 11,058 | 10 | 5 | 21 |
| Rift Valley | Kajiado | 258 | 4,121 | 2 | 1 | 3 |
| Rift Valley | Kericho | 48 | 5,972 | 4 | 2 | 8 |
| Rift Valley | Laikipia | 0 | 0 | 0 | 0 | 0 |
| Rift Valley | Nakuru | 0 | 0 | 0 | 0 | 0 |
| Rift Valley | Nandi | 36 | 4,873 | 4 | 2 | 7 |
| Rift Valley | Narok | 34 | 232 | 0 | 0 | 0 |
| Rift Valley | Samburu | 10 | 23 | 0 | 0 | 0 |
| Rift Valley | Trans Nzoia | 9 | 2,803 | 3 | 1 | 5 |
| Rift Valley | Turkana | 133 | 282 | 0 | 0 | 0 |
| Rift Valley | Uasin Gishu | 0 | 0 | 0 | 0 | 0 |
| Rift Valley | West Pokot | 162 | 2,526 | 2 | 1 | 4 |
| Western | Bungoma | 619 | 184,049 | 815 | 370 | 1555 |
| Western | Busia | 789 | 196,910 | 1672 | 766 | 3175 |
| Western | Kakamega | 1133 | 458,394 | 2387 | 1070 | 4621 |
| Western | Vihiga | 108 | 72,839 | 178 | 79 | 345 |
| **Total** |  |  | **2,239,168** | **9,344** | **4,222** | **17,962** |
